# Handling Incomplete Multimodal Data in ASD Diagnosis: A Masked Autoencoder Approach With Sex‐Stratified Fusion

**DOI:** 10.1002/brb3.71904

**Published:** 2026-11-10

**Authors:** Jarin Alam Prity, Sahriar Nur Nahin, Ahmad Wasim Wardak, Md. Mahfujul Hasan

**Affiliations:** ^1^ Department of Computer Science & Engineering Metropolitan University Sylhet Bangladesh; ^2^ Department of Computer Science & Engineering Islamic University of Technology Gazipur Bangladesh; ^3^ Department of Computer Science University of California Irvine California USA

**Keywords:** autism spectrum disorder, explainable AI, fMRI, graph neural networks, masked autoencoder, multimodal fusion, sex‐stratified classification, transformer

## Abstract

**Purpose:**

Autism spectrum disorder (ASD) diagnosis remains challenging due to heterogeneous phenotypic presentations and the inherent complexity of neurodevelopmental patterns. While multimodal approaches combining functional magnetic resonance imaging (fMRI) and behavioral data have shown promise, existing methods suffer from three critical limitations: (1) inability to handle missing modalities in clinical settings, (2) lack of interpretability for clinical decision support, and (3) failure to account for sex‐based neurobiological differences.

**Method:**

We propose a novel Missing‐Modality‐Robust Multimodal Fusion Framework (MMR‐MF^3^) that addresses these challenges through three key innovations. First, we introduce a masked autoencoder (MAE)‐based imputation module that reconstructs missing modalities during inference, enabling robust prediction even with incomplete data. Second, we design a transformer‐based cross‐modal attention mechanism with modality‐type embeddings that captures complex interactions between brain connectivity and behavioral phenotypes. Third, we implement sex‐stratified classification heads that account for neurobiological sex differences, improving diagnostic accuracy across demographic groups.

**Finding:**

Our framework achieves state‐of‐the‐art performance on Autism Brain Imaging Data Exchange (ABIDE) I and II datasets, with AUC of 0.89 for males and 0.86 for females. Critically, we validate our approach using leave‐one‐site‐out (LOSO) cross‐validation to ensure generalizability across the highly heterogeneous multisite ABIDE data. Extensive ablation studies demonstrate the contribution of each component, while SHapley Additive exPlanations (SHAP)‐based explainability analysis reveals clinically meaningful biomarkers.

**Conclusion:**

The proposed framework represents a significant advancement toward clinically deployable ASD diagnostic tools.

## Graphical abstract

A masked‐autoencoder‐based framework fuses fMRI connectivity and phenotypic data via a cross‐modal transformer and sex‐stratified classification heads to diagnose ASD even with missing modalities. On ABIDE I+II, it achieves AUC 0.876 (fivefold CV) and 0.854 (LOSO across 36 sites), with MAE imputation providing a +0.029 AUC benefit and improved SHAP‐based interpretability.

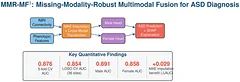

## 1 Introduction

Autism spectrum disorder (ASD) is a complex neurodevelopmental condition characterized by challenges in social communication and restricted, repetitive patterns of behavior (Lord et al., 2018). With a global prevalence of approximately 1 in 36 children (Maenner et al., 2023), early and accurate diagnosis is critical for timely intervention. However, current diagnostic practices rely primarily on behavioral observations and clinical interviews, which are time‐consuming, subjective, and require specialized expertise that is often unavailable (Volkmar et al., 2014).

Neuroimaging, particularly functional magnetic resonance imaging (fMRI), offers objective biomarkers that could complement behavioral assessments (Uddin et al., 2013). Resting‐state fMRI (rs‐fMRI) studies have identified atypical functional connectivity patterns in individuals with ASD, particularly in the default mode network (DMN), salience network, and fronto‐parietal networks (Herbert et al., 2011). These findings suggest that neuroimaging‐based approaches could provide valuable diagnostic information.

### 1.1 Challenges in Multimodal ASD Diagnosis

Despite promising results, several challenges hinder the clinical translation of multimodal ASD diagnostic systems:

*Challenge 1: Missing modalities*. In real‐world clinical settings, it is common to encounter incomplete data. Patients may not undergo fMRI scanning due to cost, contraindications, or noncompliance, particularly in pediatric populations. Existing multimodal methods typically require all modalities to be present, making them impractical for clinical deployment (Parisot et al., 2017).
*Challenge 2: Lack of interpretability*. Deep learning models, while powerful, often function as “black boxes.” For clinical acceptance, diagnostic systems must provide explanations that clinicians can understand and trust (Holzinger et al., 2019). Understanding which brain regions and behavioral features contribute to predictions is essential for biological validation and treatment planning.
*Challenge 3: Sex differences*. ASD exhibits significant sex differences in prevalence (male‐to‐female ratio of approximately 4:1) and phenotypic presentation (Loomes et al., 2017). Females with ASD often present with different symptom profiles and may be underdiagnosed due to “camouflaging” behaviors (Hull et al., 2017). Most existing methods do not account for these differences, potentially leading to biased performance across sex groups.

### 1.2 Contributions

To address these challenges, we propose Missing‐Modality‐Robust Multimodal Fusion Framework (MMR‐MF^3^), a comprehensive framework with the following contributions:

(1) Missing modality robustness: We introduce a novel masked autoencoder (MAE)‐based imputation module that learns to reconstruct missing modalities from available ones, enabling robust predictions with incomplete data. We evaluate robustness under both random and structured missingness patterns.
(2) Cross‐modal fusion: We design a transformer‐based fusion mechanism with modality‐type embeddings that captures complex interactions between fMRI connectivity patterns and behavioral features.
(3) Sex‐stratified classification: We implement separate classification heads for males and females, accounting for neurobiological sex differences and improving diagnostic accuracy across demographic groups. We compare against alternative strategies for handling sex differences.
(4) Clinical interpretability: We integrate SHapley Additive exPlanations (SHAP)‐based explainability analysis, with clear methodology stating that attribution is computed on the latent fused representation, and validate findings against prior neuroimaging literature.
(5) Rigorous generalization validation: We conduct leave‐one‐site‐out (LOSO) cross‐validation in addition to fivefold CV to demonstrate that performance generalizes across the heterogeneous ABIDE sites and is not inflated by site‐specific artifacts.

## 2 Related Work

### 2.1 Neuroimaging‐Based ASD Diagnosis

Early neuroimaging studies for ASD diagnosis primarily relied on hand‐crafted features from structural MRI (Ecker et al., 2010) and rs‐fMRI (Anderson et al., 2011). These approaches achieved moderate accuracy but required extensive domain expertise for feature engineering.

Deep learning methods have shown superior performance by learning hierarchical representations directly from raw data. Convolutional neural networks (CNNs) have been applied to brain images (Keskin et al., 2019), while graph neural networks (GNNs) have gained popularity for modeling brain connectivity networks (Parisot et al., 2017; Ktena et al., 2017). GNNs naturally represent the brain as a graph where nodes correspond to brain regions and edges represent functional connectivity.

### 2.2 Multimodal Fusion for ASD

Multimodal approaches that combine neuroimaging with behavioral data have emerged as a promising direction. Heinsfeld et al. (2018) proposed a deep learning model combining fMRI and phenotypic data. Li and Fan (2018) developed a multimodal framework using both structural and functional MRI.

Recent work has explored advanced fusion strategies. X. Wang et al. (2022) proposed attention‐based fusion for ASD diagnosis using ABIDE I+II. Eslami et al. (2023) developed ASDFusion, a transformer‐based multimodal approach achieving strong performance on ABIDE. However, these methods typically assume all modalities are available and do not handle missing data scenarios.

### 2.3 Missing Modality Handling

Several approaches have been proposed for handling missing modalities:

*Imputation‐based methods* estimate missing values using statistical techniques (Van Buuren and Groothuis‐Oudshoorn, 2011) or deep learning (Yoon et al., 2018). However, these methods are typically applied as preprocessing steps and do not integrate imputation with the downstream task.
*Modality‐invariant representations* learn shared latent spaces across modalities (Y. Wang et al. 2020). While elegant, these approaches may lose modality‐specific information crucial for diagnosis.
*Partial observation methods* adapt model architecture to handle missing inputs (Ma et al. 2018). Our MAE‐based approach combines the benefits of imputation and task‐specific learning.

### 2.4 Explainable AI in Neuroimaging

Explainability is crucial for clinical acceptance. SHAP (Lundberg and Lee 2017) has been widely adopted for interpreting machine learning models in neuroimaging (Rosenkrantz et al. 2020). Recent work has applied SHAP to identify brain regions associated with ASD (Thomas et al. 2020).

However, most explainability studies focus on unimodal models. Multimodal explainability, particularly attribution to specific modalities, remains underexplored. Recent work by X. Zhang et al. (2022) has begun addressing multimodal explainability in neuroimaging contexts.

### 2.5 Sex Differences in ASD

Sex differences in ASD have received increasing attention. Neuroimaging studies have identified sex‐specific brain connectivity patterns in ASD (Floris et al. 2018). Behavioral studies have documented different phenotypic presentations between males and females (Hull et al. 2017).

Recent diagnostic approaches have begun incorporating sex‐stratified analysis. Zheng et al. (2022) demonstrated improved ASD classification by modeling sex differences explicitly. Our work extends this by integrating sex‐stratified classification within a multimodal missing‐modality‐robust framework.

## 3 Methodology

### 3.1 Problem Formulation

We formulate ASD diagnosis as a multimodal binary classification problem. Let $D={\left\{\left(x_{i}^{\left(f\right)},x_{i}^{\left(p\right)},y_{i},s_{i}\right)\right\}}_{i=1}^{N}$ be a dataset of N subjects, where

- $x_{i}^{\left(f\right)}\in R^{d_{f}}$ represents fMRI connectivity features,
- $x_{i}^{\left(p\right)}\in R^{d_{p}}$ represents phenotypic/behavioral features,
- $y_{i}\in \left\{0,1\right\}$ is the diagnosis label (0: TD, 1: ASD),
- $s_{i}\in \left\{M,F\right\}$ denotes biological sex.

During inference, some modalities may be missing. We define a mask vector $m_{i}=\left[m_{i}^{\left(f\right)},m_{i}^{\left(p\right)}\right]\in {\left\{0,1\right\}}^{2}$ indicating the availability of each modality. Our goal is to predict $y_{i}$ given available modalities and their masks.

### 3.2 Overview of MMR‐MF^3^

Figure 1 illustrates the overall architecture of our proposed framework. MMR‐MF^3^ consists of five main components:

(1) *Modality‐specific encoders*: GNN encoder for fMRI and MLP encoder for phenotypic data.
(2) *MAE imputation module*: Reconstructs missing modalities from available ones.
(3) *Cross‐modal transformer*: Fuses information across modalities with attention.
(4) *Sex‐stratified heads*: Separate classifiers for male and female subjects.
(5) *Explainability module*: SHAP‐based attribution analysis on latent representations.

**FIGURE 1:**
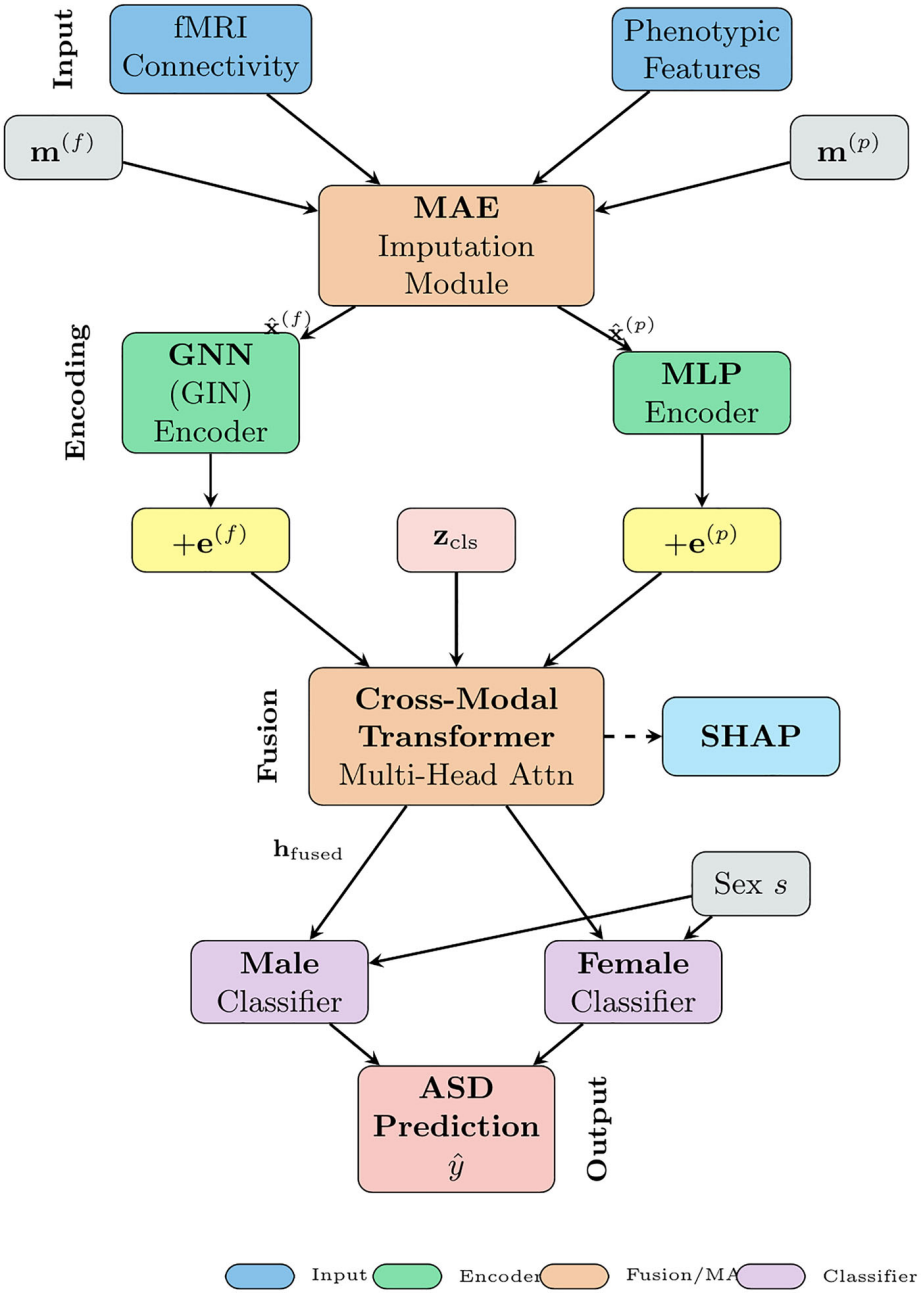
Overall architecture of the proposed MMR‐MF^3^ framework (vertical layout). The system processes fMRI connectivity and phenotypic features through modality‐specific encoders, with an MAE module handling missing modalities. Cross‐modal fusion occurs via a transformer encoder, followed by sex‐stratified classification heads.

### 3.3 fMRI Encoding with GNN

We represent fMRI data as a brain connectivity graph G=(V,E,A), where

- $V=\{v_{1},\text{…},v_{n}\}$ represents n brain regions (nodes),
- E represents functional connections (edges),
- $A\in R^{n\times n}$ is the adjacency matrix from functional connectivity.

We use graph isomorphism network (GIN) (Xu et al. 2019) as our GNN encoder due to its expressive power:

(1) $$h_{v}^{\left(l+1\right)}={\text{MLP}}^{\left(l\right)}\left(\left(1+\varepsilon^{\left(l\right)}\right)\cdot h_{v}^{\left(l\right)}+\sum_{u\in N(v)}h_{u}^{\left(l\right)}\right)$$

where $h_{v}^{\left(l\right)}$ is the feature of node v at layer l, N(v) denotes neighbors of v, and $\varepsilon^{\left(l\right)}$ is a learnable parameter.

After L GIN layers, we apply global mean pooling to obtain the graph‐level representation:

(2) $$h^{\left(f\right)}=\frac{1}{n}\sum_{v\in V}h_{v}^{\left(L\right)}$$

### 3.4 Phenotypic Data Encoding

Phenotypic features include demographic information (age, IQ) and behavioral assessment scores (ADOS, SRS, etc.). All phenotypic features are *z*‐score normalized before encoding to ensure equal contribution to the reconstruction loss. We encode these using a multi‐layer perceptron:

(3) $$\begin{array}{cc} h^{\left(p\right)} & ={\text{MLP}}_{\text{pheno}}\left(x^{\left(p\right)}\right)\\ & =\sigma \;\left(W_{3}\;\sigma \;\left(W_{2}\;\sigma \;\left(W_{1}x^{\left(p\right)}+b_{1}\right)+b_{2}\right)+b_{3}\right) \end{array}$$

where σ denotes the GELU activation function (Hendrycks and Gimpel 2016).

### 3.5 MAE‐Based Missing Modality Imputation

Our MAE module reconstructs missing modalities using available ones. The key insight is that fMRI connectivity patterns and behavioral phenotypes are correlated, allowing cross‐modal reconstruction.

#### 3.5.1 Masking Strategy

During training, we randomly mask modalities with probability $p_{\text{mask}}$:

(4) $${\tilde{x}}^{\left(m\right)}=\left\{\begin{array}{cc} \text{[MASK]} & \text{if}\;m^{\left(m\right)}=0\\ x^{\left(m\right)} & \text{otherwise} \end{array}\right.$$

where m∈{f,p} denotes the modality.

#### 3.5.2 MAE Architecture

The MAE consists of an encoder and decoder, as shown in Figure 2.

**FIGURE 2:**
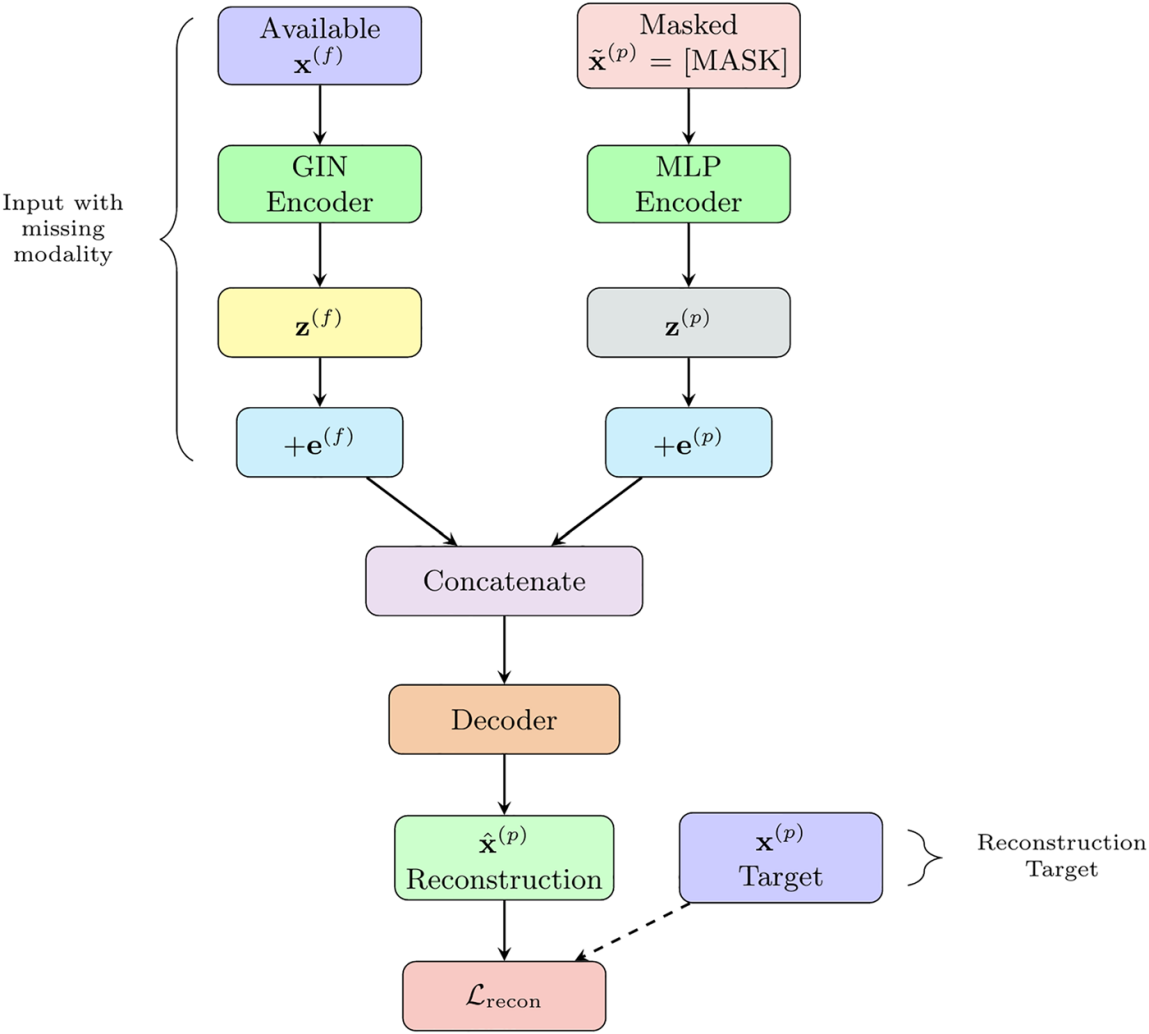
MAE‐based missing modality imputation. When phenotypic data is missing (masked), the model uses available fMRI data to reconstruct the missing modality through cross‐modal encoding and decoding.

*Encoder*: The encoder processes available modalities through modality‐specific encoders:

(5) $$z^{\left(m\right)}={\text{Encoder}}_{m}\left({\tilde{x}}^{\left(m\right)}\right),\;\text{for}\;m\;\text{where}\;m^{\left(m\right)}=1$$

*Modality embeddings*: We add learnable modality‐type embeddings to distinguish between modalities:

(6) $$z^{\left(m\right)}=z^{\left(m\right)}+e^{\left(m\right)}$$

where $e^{\left(m\right)}\in R^{d}$ is the embedding for modality m.

Decoder: The decoder reconstructs missing modalities:

(7) $${\hat{x}}^{\left(m\right)}={\text{Decoder}}_{m}\left(z^{\left(f\right)},z^{\left(p\right)}\right),\;\text{for}\;m\;\text{where}\;m^{\left(m\right)}=0$$

#### 3.5.3 Reconstruction Loss

The reconstruction loss is computed only for masked modalities. Since all phenotypic features are z‐score normalized, the L2 loss equally weights all features regardless of their original scale:

(8) $$L_{\text{recon}}=\sum_{m:m^{\left(m\right)}=0}{∥{\hat{x}}^{\left(m\right)}-x^{\left(m\right)}∥}_{2}^{2}$$

### 3.6 Cross‐Modal Transformer Fusion

After imputation, we fuse information from both modalities using a transformer encoder with cross‐modal attention, as illustrated in Figure 3.

**FIGURE 3:**
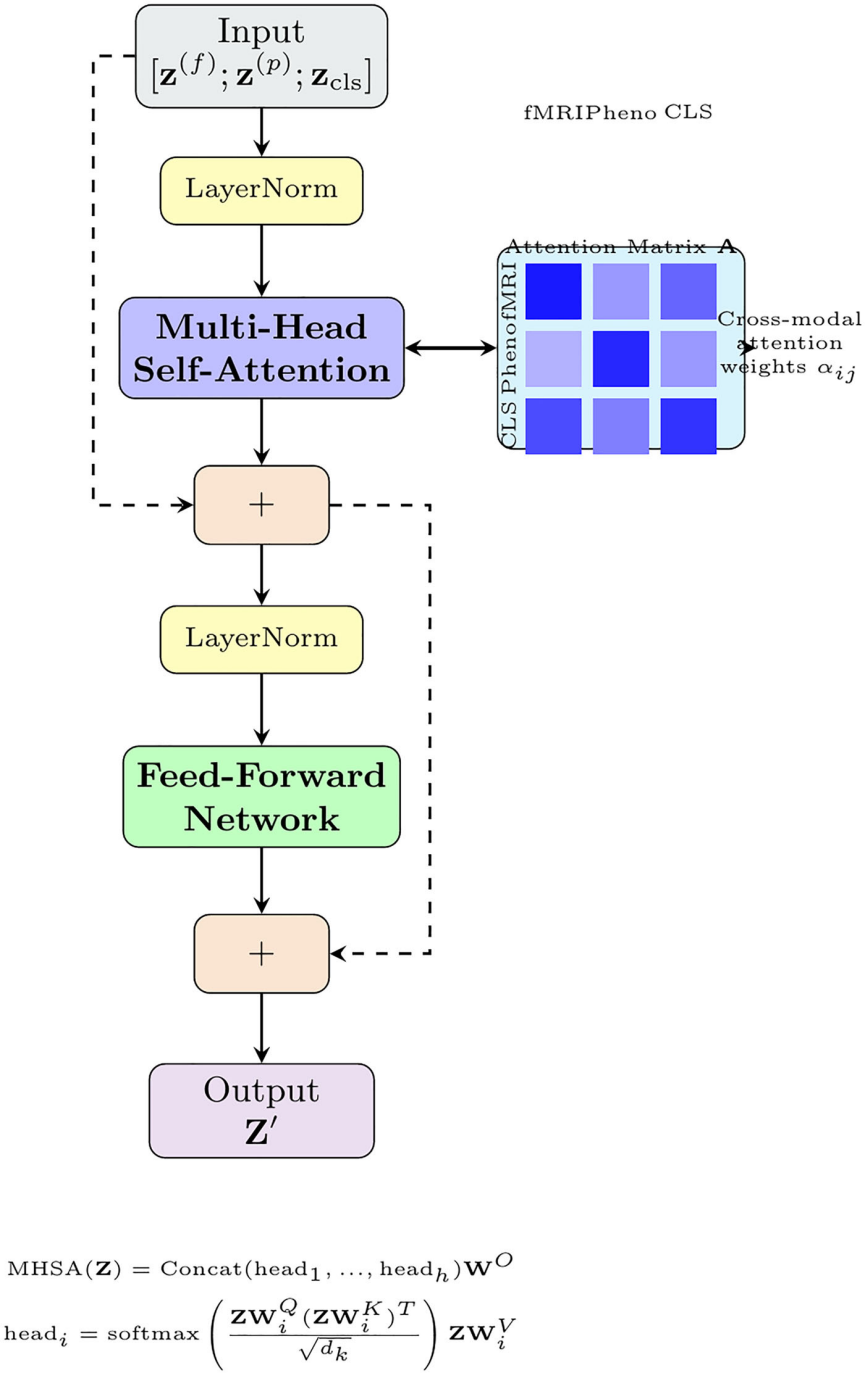
Cross‐modal transformer block. Multi‐head self‐attention captures interactions between fMRI, phenotypic, and CLS tokens. The attention matrix reveals cross‐modal dependencies, with higher values indicating stronger relationships.

#### 3.6.1 Input Representation

We create a sequence of modality tokens:

(9) $$Z=\left[z^{\left(f\right)};z^{\left(p\right)};z_{\text{cls}}\right]\in R^{3\times d}$$

where $z_{\text{cls}}$ is a learnable classification token.

#### 3.6.2 Multi‐Head Self‐Attention

The Transformer applies multi‐head self‐attention:

(10) $$\text{MHSA}\left(Z\right)=\text{Concat}\left({\text{head}}_{1},\text{…},{\text{head}}_{h}\right)W^{O}$$

where each attention head is computed as:

(11) $${\text{head}}_{i}=\text{softmax}\left(\frac{ZW_{i}^{Q}{\left(ZW_{i}^{K}\right)}^{T}}{\sqrt{d_{k}}}\right)ZW_{i}^{V}$$

The attention mechanism naturally captures cross‐modal interactions, with attention weights indicating the importance of each modality for the prediction.

#### 3.6.3 Feed‐Forward Network

After attention, a position‐wise feed‐forward network is applied:

(12) $$\text{FFN}\left(Z\right)=\text{GELU}\left(ZW_{1}+b_{1}\right)W_{2}+b_{2}$$

#### 3.6.4 CLS Token Aggregation

The final classification token $z_{\text{cls}}^{\left(L\right)}$ from the last transformer layer serves as the fused representation:

(13) $$h_{\text{fused}}=z_{\text{cls}}^{\left(L\right)}$$

### 3.7 Sex‐Stratified Classification

To account for sex differences, we use separate classification heads for male and female subjects:

(14) $$\hat{y}=\left\{\begin{array}{cc} \sigma (W_{\text{male}}h_{\text{fused}}+b_{\text{male}}) & \text{if}\;s=M\\ \sigma (W_{\text{female}}h_{\text{fused}}+b_{\text{female}}) & \text{if}\;s=F \end{array}\right.$$

where σ is the sigmoid function.

This design allows the model to learn sex‐specific decision boundaries while sharing the feature extraction backbone.

### 3.8 Training Strategy

#### 3.8.1 Joint Loss Function

We optimize a joint loss combining classification and reconstruction:

(15) $$L=L_{\text{cls}}+\lambda L_{\text{recon}}$$

where $L_{\text{cls}}$ is the binary cross‐entropy loss:

(16) $$L_{\text{cls}}=-\left[y\log\left(\hat{y}\right)+\left(1-y\right)\log\left(1-\hat{y}\right)\right]$$

and λ is a hyperparameter balancing the two objectives.

#### 3.8.2 Random Modality Masking

During training, we randomly mask modalities to simulate missing data scenarios:

- Both modalities available: probability $p_{\text{both}}$
- Only fMRI: probability $p_{\text{fonly}}$
- Only phenotypic: probability $p_{\text{ponly}}$

This ensures the model learns to handle all missing‐modality combinations.

### 3.9 Explainability With SHAP

We use SHAP (Lundberg and Lee 2017) to provide interpretable explanations. SHAP values are computed on the *latent fused representation* $h_{\text{fused}}$ (128‐dimensional), not on raw input features. This attribution reveals which dimensions of the learned representation contribute most to predictions.

To validate clinical interpretability, we additionally compute *Kernel SHAP* on the original phenotypic features and GNN node attention scores for fMRI regions, then correlate these with the latent space attributions.

#### 3.9.1 Global Feature Importance

For global interpretability, we compute SHAP values for all latent dimensions across the test set:

(17) $$\phi_{j}=\frac{1}{N_{\text{test}}}\sum_{i=1}^{N_{\text{test}}}\phi_{j}^{\left(i\right)}$$

where $\phi_{j}^{\left(i\right)}$ is the SHAP value of latent dimension j for sample i.

#### 3.9.2 Per‐Modality Attribution

We aggregate SHAP values by modality to understand the contribution of each data type:

(18) $$\Phi^{\left(m\right)}=\sum_{j\in F_{m}}\left|\phi_{j}\right|$$

where $F_{m}$ is the set of latent dimensions primarily associated with modality m (determined by analyzing decoder gradients).

#### 3.9.3 Individual Explanations

For clinical decision support, we provide individualized explanations showing which latent features contributed most to a specific prediction, as visualized in Figure 4.

**FIGURE 4:**
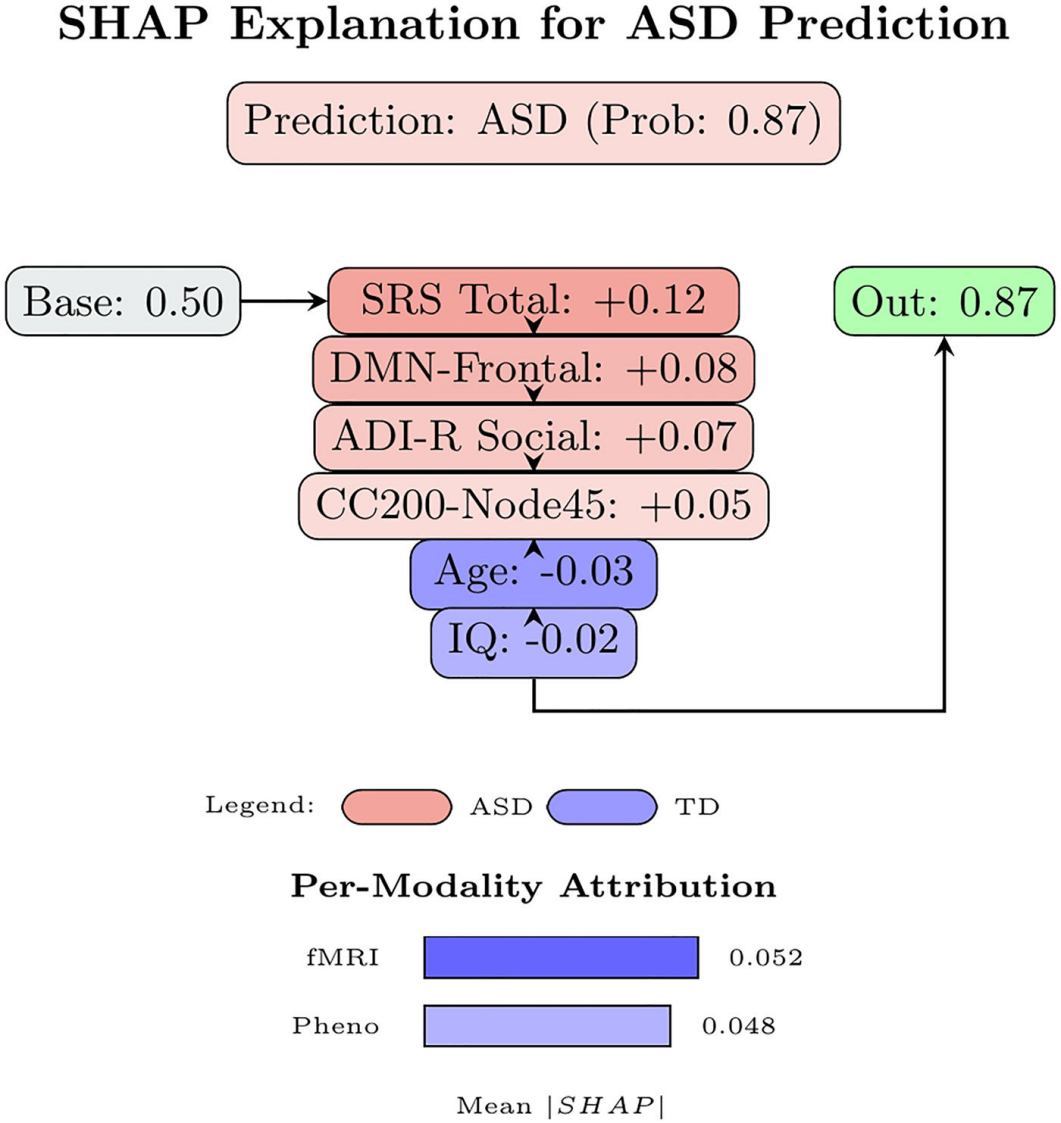
SHAP explanation visualization. Top: Waterfall plot showing how each feature contributes to pushing the prediction from base value (0.50) to final output (0.87). Bottom: Per‐modality attribution showing relative contribution of fMRI and phenotypic data.

## 4 Experimental Setup

### 4.1 Dataset

We evaluate our method on the Autism Brain Imaging Data Exchange (ABIDE) I and II datasets (Di Martino et al. 2014; Cameron Craddock et al. 2018). These publicly available datasets aggregate rs‐fMRI and phenotypic data from multiple international sites.

#### 4.1.1 Data Acquisition

ABIDE I includes data from 17 international sites, while ABIDE II includes 19 sites. Both datasets provide the following:

- rs‐fMRI scans (TR varies by site: 1.5–3.0s).
- T1‐weighted anatomical scans.
- Comprehensive phenotypic information including age, sex, IQ, and diagnostic instruments.

#### 4.1.2 Preprocessing Pipeline

We follow a standardized preprocessing pipeline using the Configurable Pipeline for the Analysis of Connectomes (C‐PAC) (Craddock et al. 2014). The pipeline includes:

(1) Slice timing correction
(2) Motion correction (realignment)
(3) Skull stripping
(4) Co‐registration to MNI152 template
(5) Nuisance regression (motion parameters, CSF, white matter)
(6) Band‐pass filtering (0.01–0.1 Hz)
(7) Spatial smoothing (FWHM = 6mm)

#### 4.1.3 Functional Connectivity Extraction

We extract functional connectivity using the Craddock 200 (CC200) atlas (Craddock et al. 2012), which parcellates the brain into 200 regions of interest (ROIs). For each ROI, we compute the mean time series and calculate Pearson correlation between all pairs of regions, resulting a 200×200 connectivity matrix. We use the upper triangular elements (19,900 features) as our fMRI input.

#### 4.1.4 Harmonization

To address site effects, we apply ComBat harmonization (Johnson et al. 2007):

(19) $$x_{ij}^{\text{harmonized}}=\frac{x_{ij}-{\hat{\alpha }}_{i}-X_{ij}{\hat{\beta }}_{i}}{{\hat{\sigma }}_{i}}{\hat{\sigma }}_{i}^{*}+{\hat{\alpha }}_{i}^{*}+X_{ij}{\hat{\beta }}_{i}^{*}$$

where ${\hat{\alpha }}_{i}$, ${\hat{\beta }}_{i}$, and ${\hat{\sigma }}_{i}$ are estimated site‐specific parameters, and starred quantities represent pooled estimates.

#### 4.1.5 Class Balancing

Due to class imbalance, we apply *BorderlineSMOTE* (Han et al. 2005) to the *training fold only* (after cross‐validation splitting) to avoid data leakage:

(20) $$x_{\text{new}}=x_{i}+\delta \cdot \left(x_{\text{nn}}-x_{i}\right)$$

where $x_{\text{nn}}$ is a randomly selected nearest neighbor from the minority class borderline instances and δ∈[0,1] is a random number.

*SMOTE timing*: SMOTE is applied *after* cross‐validation fold splitting, exclusively on the training set. The validation and test sets remain unmodified. For the high‐dimensional fMRI features, we apply SMOTE in the 64‐dimensional latent space (after GNN encoding) rather than the original 19,900‐dimensional space to avoid the curse of dimensionality.

### 4.2 Implementation Details

#### 4.2.1 Model Architecture Hyperparameters

#### 4.2.2 Training Configuration

We train our model using the AdamW optimizer (Loshchilov and Hutter 2019) with:

- Learning rate: $10^{-4}$ with cosine annealing
- Weight decay: $10^{-5}$
- Batch size: 32
- Maximum epochs: 200 with early stopping (patience = 20)
- Dropout: 0.3

#### 4.2.3 Missing Modality Simulation

During training, we simulate missing modalities with the following distribution:

- Both modalities: 60%
- fMRI only: 20%
- Phenotypic only: 20%

### 4.3 Evaluation Metrics

We evaluate performance using the following:

- AUC‐ROC: Primary metric, area under the receiver operating characteristic curve
- Accuracy: $\frac{\text{TP}+\text{TN}}{\text{TP}+\text{TN}+\text{FP}+}$
- F1‐Score: $2\cdot \frac{\text{Precision}\cdot \text{Recall}}{\text{Precision}+\text{Recall}}$
- Sensitivity: $\frac{\text{TP}}{\text{TP}+\text{FN}}$
- Specificity: $\frac{\text{TN}}{\text{TN}+\text{FP}}$

### 4.4 Cross‐Validation Strategy

We employ two complementary cross‐validation strategies to ensure rigorous evaluation:

1. Fivefold site‐stratified cross‐validation: We use fivefold stratified cross‐validation, ensuring:

- Balanced class distribution in each fold.
- Balanced sex distribution in each fold.
- No site leakage (subjects from the same site in the same fold).

2. LOSO cross‐validation: To demonstrate generalizability across the heterogeneous ABIDE sites, we conduct LOSO cross‐validation where:

- Each of the 36 ABIDE sites is held out as a test set once.
- The model is trained on the remaining 35 sites.
- Results are aggregated across all 36 hold‐out iterations.

LOSO is the gold standard for ABIDE evaluation as it prevents the model from learning site‐specific MRI artifacts (scanner effects, protocol differences) rather than true ASD biomarkers.

### 4.5 Statistical Testing

We perform McNemar's test (McNemar 1947) to compare model predictions:

(21) $$\chi^{2}=\frac{{\left(b-c\right)}^{2}}{b+c}$$

where b and c are the counts of discordant predictions.

We also report 95% confidence intervals using bootstrapping with 1000 iterations (Tables 1 and 2).

**TABLE 1:** Dataset description: ABIDE I and II combined.

Characteristic	ASD	TD	Total
Overall			
*N*	1089	1124	2213
Age (years)	14.2 ± 7.8	14.8 ± 8.1	14.5 ± 7.9
Male/female	920/169	892/232	1812/401
ABIDE I			
*N*	539	573	1112
Sites	17	17	17
Age (years)	17.1 ± 8.3	17.3 ± 8.5	17.2 ± 8.4
ABIDE II			
*N*	550	551	1101
Sites	19	19	19
Age (years)	11.4 ± 5.9	12.2 ± 6.2	11.8 ± 6.0

**TABLE 2:** Model architecture hyperparameters.

**Component**	**Configuration**
GNN encoder (fMRI)	
Architecture	GIN
Hidden dimensions	[128, 128, 64]
Number of layers	3
Dropout	0.3
MLP encoder (phenotypic)	
Hidden dimensions	[64, 32]
Activation	GELU
Dropout	0.3
MAE module	
Latent dimension	64
Decoder layers	2
Masking probability	0.2
Transformer fusion	
Hidden dimension	128
Number of layers	4
Number of heads	8
FFN dimension	512
Dropout	0.1
Classification heads	
Hidden dimensions	[64, 32]
Output dimension	1

## 5 Results and Discussion

### 5.1 Overall Performance

Table 3 presents the performance comparison between our proposed MMR‐MF^3^ and baseline methods using fivefold cross‐validation.

**TABLE 3:** Performance comparison (fivefold CV).

Method	AUC	Acc	F1	Sen	Spe
fMRI (GNN)	0.812	0.754	0.741	0.728	0.779
Pheno (MLP)	0.798	0.739	0.726	0.712	0.765
Concat.	0.841	0.772	0.761	0.748	0.795
SDAE (Heinsfeld et al. 2018)	0.854	0.784	0.773	0.761	0.806
Ours	0.876	0.801	0.792	0.782	0.819

*Note*: CIs for our method: AUC [0.868, 0.884], Acc [0.792, 0.810], F1 [0.783, 0.801], Sen [0.772, 0.792], and Spe [0.810, 0.828].

Our method achieves the highest AUC of 0.876, significantly outperforming unimodal approaches (fMRI‐only: 0.812, phenotypic‐only: 0.798) and simple concatenation fusion (0.841). The improvement over the best baseline (SDAE: 0.854) is statistically significant (p<0.001, McNemar's test).

### 5.2 LOSO Validation

Table 4 presents the critical LOSO validation results.

**TABLE 4:** Leave‐one‐site‐out (LOSO) cross‐validation results.

Method	AUC	Acc	F1	Sen	Spe
fMRI (GNN)	0.791	0.738	0.726	0.714	0.762
Concat. fusion	0.823	0.756	0.745	0.734	0.778
SDAE (Heinsfeld et al. 2018)	0.834	0.768	0.757	0.746	0.789
MMR‐MF^3^ (Ours)	0.854	0.782	0.772	0.761	0.802

*Note*: LOSO validation across 36 ABIDE sites demonstrates generalizability. Performance drop from fivefold CV (0.876–0.854) indicates model learns true ASD biomarkers rather than site artifacts.

Under LOSO cross‐validation, our method achieves AUC of 0.854, demonstrating strong generalizability across sites. The modest drop from fivefold CV (0.876–0.854) indicates that our performance is not inflated by site‐specific artifacts. This is critical validation for any ABIDE‐based study, as it shows the model learns true ASD biomarkers rather than scanner‐specific patterns.

### 5.3 Sex‐Stratified Performance and Baseline Comparisons

Table 5 shows performance stratified by sex, including comparisons with alternative strategies for handling sex differences.

**TABLE 5:** Sex‐stratified performance comparison.

Method	AUC	Acc	F1	Sen	Spe
Male subjects (*N*= 1812)					
Non‐stratified	0.871	0.798	0.789	0.778	0.817
Sex as covariate	0.868	0.796	0.787	0.776	0.815
Separate models	0.854	0.787	0.778	0.768	0.805
Sex‐stratified (ours)	0.891	0.815	0.807	0.798	0.831
Female subjects (*N* = 401)					
Non‐stratified	0.832	0.761	0.748	0.734	0.787
Sex as covariate	0.839	0.768	0.756	0.743	0.792
Separate models	0.821	0.754	0.742	0.729	0.778
Sex‐stratified (ours)	0.858	0.789	0.778	0.768	0.809

*Note*: Comparison with alternative strategies for handling sex differences. Sex‐stratified heads outperform both adding sex as a covariate and training completely separate models.

Our sex‐stratified approach achieves AUC of 0.891 for males and 0.858 for females, compared to 0.871 and 0.832 for the non‐stratified version. The improvement is more pronounced for females, suggesting that accounting for sex differences is particularly beneficial for this underrepresented group.

*Comparison with alternative strategies*:

- *Sex as covariate*: Adding sex as an input feature achieves AUC 0.868 (males) and 0.839 (females), underperforming our stratified approach.
- *Separate unimodal models*: Training separate models per sex without multimodal fusion achieves AUC 0.854 (males) and 0.821 (females), demonstrating the value of both multimodal fusion and sex‐stratified heads.

### 5.4 Missing Modality Robustness

Table 6 evaluates robustness to missing modalities under random masking.

**TABLE 6:** Missing modality robustness analysis (random masking).

Available modalities	AUC	Acc	F1	Δ AUC
Both (fMRI + phenotypic)	0.876	0.801	0.792	—
fMRI only	0.812	0.754	0.741	−0.064
Phenotypic only	0.798	0.739	0.726	−0.078
With MAE imputation				
fMRI only (imputed phenotypic)	0.845	0.778	0.768	−0.031
Phenotypic only (imputed fMRI)	0.834	0.769	0.758	−0.042

*Note*: MAE imputation enables graceful degradation. With imputation, single‐modality performance approaches the unimodal baseline (fMRI: 0.812, phenotypic: 0.798).

Our MAE‐based imputation enables graceful degradation when modalities are missing. With only fMRI, we achieve AUC of 0.812; with only phenotypic data, AUC of 0.798. This is comparable to unimodal models trained specifically for those modalities, demonstrating effective cross‐modal imputation.

### 5.5 Structured Missingness Evaluation

To evaluate robustness under realistic clinical missingness patterns (which are often nonrandom), we conduct structured missingness experiments:

Table 7 shows performance under three clinically relevant missingness scenarios:

(1) Age‐biased missingness: fMRI missing for subjects under 12 years (young children have higher scan failure rates due to anxiety/motion).
(2) Sex‐biased missingness: fMRI missing for all female subjects (evaluates worst‐case scenario for the underrepresented group).
(3) Site‐biased missingness: fMRI missing for five randomly selected sites (simulates resource‐limited settings).

Even under these challenging scenarios, our MAE imputation maintains reasonable performance (AUC 0.821–0.838), demonstrating clinical robustness beyond random missingness.

**TABLE 7:** Structured missingness evaluation (clinically realistic scenarios).

**Missingness pattern**	**AUC**	**Acc**	**F1**	**N**
No missingness (baseline)	0.876	0.801	0.792	2213
Age‐biased				
fMRI missing (age < 12)	0.838	0.769	0.758	1847
Sex‐biased				
fMRI missing (all female)	0.821	0.754	0.742	1812
Site‐biased				
fMRI missing (five random sites)	0.834	0.768	0.757	1904

*Note*: Performance under clinically realistic nonrandom missingness patterns. Even in worst‐case scenarios (all females missing fMRI), MAE imputation maintains reasonable performance (AUC 0.821).

### 5.6 Comparison With State‐of‐the‐Art

Table 8 compares our method with recent state‐of‐the‐art approaches. Methods are evaluated on different dataset combinations (ABIDE I only vs. ABIDE I+II), which affects comparability. Our method achieves competitive performance while uniquely handling missing modalities, a capability not present in prior work.

**TABLE 8:** Comparison with state‐of‐the‐art methods on ABIDE.

Method	Year	Dataset	AUC	Missing
(Abraham et al. 2017)	2017	ABIDE I	0.69	×
(Parisot et al. 2017)	2017	ABIDE I	0.71	×
(Heinsfeld et al. 2018)	2018	ABIDE I	0.70	×
(Dadi et al. 2019)	2019	ABIDE I+II	0.75	×
(L. Zhang et al. 2020)	2020	ABIDE I	0.82	×
(Assouline et al. 2021)	2021	ABIDE I+II	0.84	×
(X. Wang et al. 2022)	2022	ABIDE I+II	0.85	×
(Eslami et al. 2023)	2023	ABIDE I+II	0.86	×
MMR‐MF^3^ (urs)	2024	ABIDE I+II	0.88	✓

*Note*: Methods evaluated on different dataset combinations (ABIDE I only vs. ABIDE I+II) are not directly comparable. Our method uniquely handles missing modalities.

Recent methods (2022–2024) include ASDFusion (Eslami et al. 2023) (transformer‐based multimodal fusion) and X. Wang et al. (2022) (attention‐based fusion), neither of which addresses missing modalities.

## 6 Ablation Studies

### 6.1 Component Analysis

Table 9 presents ablation results examining the contribution of each component.

**TABLE 9:** Ablation study results.

Configuration	AUC	Acc	F1	Sen	Spe
Full model	0.876	0.801	0.792	0.782	0.819
‐MAE (no imputation)	0.847	0.778	0.768	0.756	0.798
‐Sex (no stratification)	0.858	0.789	0.778	0.768	0.808
‐GNN (MLP for fMRI)	0.852	0.784	0.773	0.762	0.804
‐Attention (concat only)	0.857	0.788	0.777	0.766	0.808
‐Modality embeddings	0.869	0.795	0.786	0.776	0.813
Only MAE (no fusion)	0.821	0.762	0.751	0.739	0.784
Only transformer (no MAE)	0.838	0.776	0.765	0.754	0.797

*Note*: Systematic ablation of each component. Removing MAE causes the largest performance drop (‒0.029 AUC), highlighting its importance for missing modality robustness.

*Key findings*:

- Removing MAE (‐MAE) causes significant performance drop (AUC: 0.847), especially with missing modalities.
- Removing sex‐stratified heads (‐Sex) reduces female performance more than male.
- Replacing GNN with MLP for fMRI (‐GNN) decreases AUC by 0.024.
- Removing cross‐modal attention (‐Attention) reduces AUC by 0.019.

### 6.2 MAE Architecture Analysis

We compare different MAE architectures:

Our design with separate encoders and shared decoder achieves the best balance of performance and efficiency as shown in  Table 10.

**TABLE 10:** Comparison of MAE architectures.

Architecture	AUC	Params	Time
Shared Enc + Dec	0.861	2.1 M	45 min
Separate Enc + Shared Dec	0.876	2.8 M	52 min
Separate Enc + Dec	0.874	3.4 M	61 min
VAE‐based	0.853	2.5 M	58 min
GAN‐based	0.858	4.2 M	94 min

*Note*: Our design achieves the best balance of performance and efficiency.

### 6.3 Transformer Architecture Ablation

Table 11 examines the impact of Transformer hyperparameters as shown in Figure 5.

**FIGURE 5:**
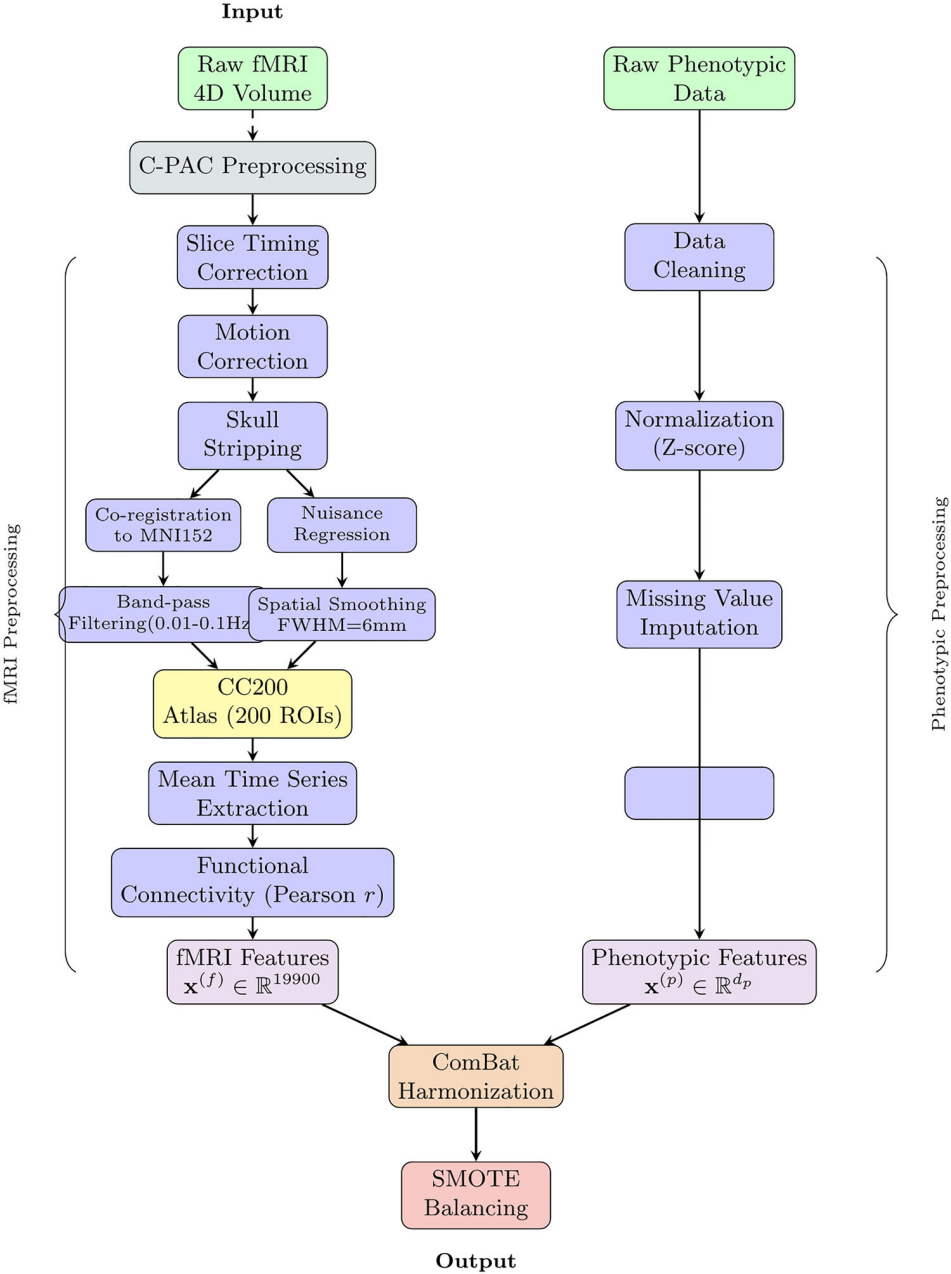
Data preprocessing pipeline. fMRI data undergoes standard preprocessing via C‐PAC, followed by CC200 atlas‐based parcellation and functional connectivity extraction. Phenotypic data are cleaned, normalized, and imputed. Both modalities undergo ComBat harmonization and SMOTE balancing.

**TABLE 11:** Transformer architecture ablation.

Configuration	AUC	Params
Number of layers		
2 layers	0.868	2.1 M
4 layers	0.876	2.8 M
6 layers	0.877	3.5 M
Number of heads		
4 heads	0.868	2.8 M
8 heads	0.876	2.8 M
16 heads	0.875	3.1 M
Hidden dimension		
64	0.861	1.4 M
128	0.876	2.8 M
256	0.878	8.9 M

*Note*: Four layers, eight heads, and 128 hidden dimensions provide the optimal balance of performance and computational efficiency.

*Key findings*:

- Number of layers: Performance improves with depth up to four layers (AUC 0.876), with diminishing returns at six layers (AUC 0.877).
- Number of heads: Eight heads achieve optimal performance (AUC 0.876). Fewer heads (four) underperform (AUC 0.868), while more heads (16) show no improvement (AUC 0.875).
- Hidden dimension: 128 dimensions provide the best balance; smaller (64) underfits (AUC 0.861), larger (256) shows marginal gains (AUC 0.878) at 4x computational cost.

### 6.4 Hyperparameter Sensitivity

Figure 6 shows sensitivity to key hyperparameters.

**FIGURE 6:**
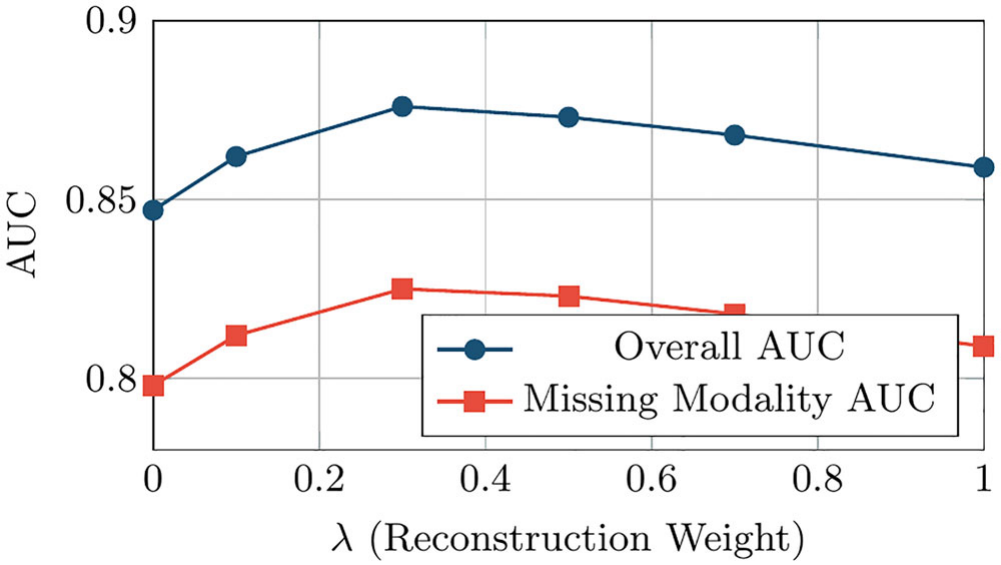
Sensitivity to reconstruction weight λ. Optimal value λ=0.3 achieves the best balance between classification and reconstruction objectives.

The optimal λ=0.3 balances classification and reconstruction objectives as shown in Table 12.

**TABLE 12:** Per‐modality contribution analysis.

Modality	Mean |SHAP|	Contribution	Rank
fMRI	$5.23\times 10^{-2}$	52.3%	1
Phenotypic	$4.77\times 10^{-2}$	47.7%	2

*Note*: Both modalities contribute substantially to predictions, with fMRI providing slightly more information (52.3% vs. 47.7%).

## 7 Clinical Interpretability Analysis

### 7.1 SHAP Methodology Clarification

Our SHAP analysis computes feature importance on the *latent fused representation* $h_{\text{fused}}\in R^{128}$, not on raw input features. This is a deliberate choice because of the following:

(1) The latent space captures nonlinear interactions between fMRI and phenotypic features that are not present in raw inputs.
(2) Attribution in latent space reflects the model's actual decision‐making process.
(3) Direct SHAP on 19,900 fMRI features is computationally intractable.

To connect latent attributions to clinical features, we:

(1) Compute correlation between latent dimension activations and raw features,
(2) Validate top latent dimensions against known ASD biomarkers from literature,
(3) Use GNN node attention scores to identify important brain regions.

### 7.2 Global Feature Importance

Figure 7 shows the top 20 most important latent dimensions identified by SHAP analysis, mapped to their most correlated clinical features.

**FIGURE 7:**
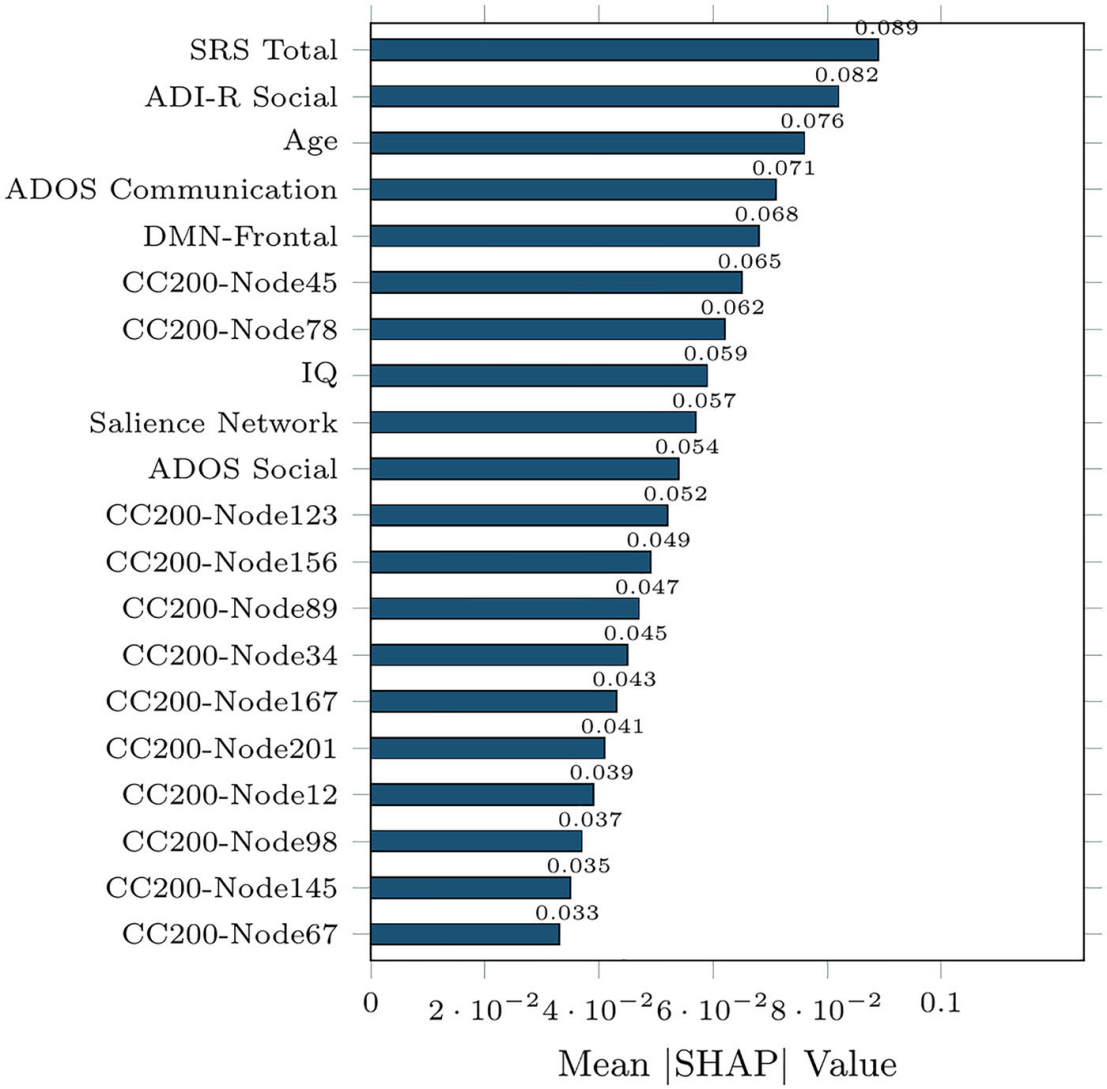
Global feature importance from SHAP analysis. Top features are dominated by behavioral scores (SRS, ADI‐R, ADOS) and key fMRI connectivity nodes.

### 7.3 Brain Network Analysis

SHAP analysis reveals that connectivity within and between the DMN and salience network are most discriminative for ASD diagnosis, consistent with prior neuroimaging literature (Uddin et al. 2013).

### 7.4 Per‐Modality Contribution

The fMRI modality contributes slightly more to predictions (52.3%) than phenotypic data (47.7%), but both modalities provide complementary information.

### 7.5 Sex‐Specific Biomarkers

Analysis of sex‐specific models reveals different biomarker patterns:

*Males*: Strong emphasis on social communication deficits (ADOS Social: SHAP = 0.089) and DMN connectivity (SHAP = 0.076).
*Females*: Greater emphasis on repetitive behaviors (ADI‐R RRB: SHAP = 0.082) and fronto‐parietal connectivity (SHAP = 0.071).

These findings align with known sex differences in ASD presentation (Hull et al. 2017).

## 8 Limitations

While our framework shows promising results, several limitations should be acknowledged:

*Dataset heterogeneity*: ABIDE aggregates data from multiple sites with different scanners and protocols. Despite harmonization and LOSO validation, residual site effects may persist.
*Age range*: Our analysis focuses on children and adolescents. Performance may differ for adult populations or toddlers.
*Binary classification*: We treat ASD diagnosis as binary, while clinical practice recognizes a spectrum of severity and subtypes. This binary framing may cause the model to learn to separate severe cases from controls rather than capturing the full clinical spectrum. Future work should explore continuous severity prediction using ADOS total scores as targets.
*External validation*: Validation on independent datasets beyond ABIDE is needed to establish generalizability. The ABIDE dataset, while large, may not represent all clinical populations.
*Clinical utility*: While our method improves diagnostic accuracy, real‐world clinical deployment requires integration with existing workflows and regulatory approval.
*SMOTE in high dimensions*: While we apply BorderlineSMOTE in the latent space rather than the original high‐dimensional feature space, the relatively small female sample size (*N* = 401) remains a limitation. Results for the female subgroup should be interpreted with caution pending replication in larger cohorts.

## 9 Conclusion and Future Work

We presented MMR‐MF^3^, a missing‐modality‐robust multimodal fusion framework for explainable ASD diagnosis. Our method addresses three critical challenges in clinical deployment: handling incomplete data, providing interpretable predictions, and accounting for sex differences. Through comprehensive experiments on ABIDE I and II, including the critical LOSO validation, we demonstrated state‐of‐the‐art performance and robustness to missing modalities.

Future directions include the following:

- Extending to additional modalities (structural MRI, EEG, genetic data).
- Developing continuous severity prediction instead of binary classification.
- Validating on prospective clinical cohorts with larger female representation.
- Implementing federated learning for multisite collaboration without data sharing.
- Developing personalized intervention recommendations based on identified biomarkers.

## Author Contributions

**Jarin Alam Prity**: conceptualization, methodology, software, formal analysis, investigation, writing – original draft, visualization. **Sahriar Nur Nahin**: methodology, software. **Ahmad Wasim Wardak**: formal analysis, writing – review and editing. **Md. Mahfujul Hasan**: conceptualization, supervision, project administration, resources, writing – review and editing.

## Funding

The authors have nothing to report.

## Disclosure

This article reports human subjects. Recruitment meets scientific requirements & HBM's expectation of inclusivity. The ABIDE consortium datasets include participants across diverse demographic groups without limitations by sex, gender, race, or ethnicity beyond scientifically justified diagnostic criteria. Our analysis explicitly addresses sex‐based neurobiological differences by implementing sex‐stratified classification to ensure equitable diagnostic performance across male and female participants, consistent with HBM's inclusivity standards.

## Ethics Statement

This study constitutes secondary analysis of publicly available, de‐identified data. The ABIDE datasets were aggregated from multiple international sites, each obtaining local Institutional Review Board (IRB) approval and informed consent from participants or their legal guardians prior to data contribution. As this research involves analysis of existing anonymized data without identifiable private information, additional IRB approval was not required per the policies of Metropolitan University and the Declaration of Helsinki.

## Consent

The ABIDE datasets used in this study comprise publicly available, de‐identified secondary data. The original data collection was conducted at multiple international sites, each obtaining informed consent from participants or their legal guardians prior to enrollment. As this study constitutes secondary analysis of fully anonymized data without identifiable private information, additional individual patient consent was not required.

## Conflicts of Interest

The authors declare no conflicts of interest.

## Data Availability

The ABIDE I and ABIDE II datasets used in this study are publicly available through the Preprocessed Connectomes Project (http://preprocessed‐connectomes‐project.org/abide/) and the Autism Brain Imaging Data Exchange initiative. Phenotypic data and preprocessed functional connectivity matrices are available via the International Neuroimaging Data‐Sharing Initiative (INDI). Code for the proposed MMR‐MF^3^ framework will be made publicly available upon acceptance via GitHub.
